# Structures Controlled by Entropy: The Flexibility of Strychnine as Example

**DOI:** 10.3390/molecules27227987

**Published:** 2022-11-17

**Authors:** Ulrich Sternberg, Raiker Witter

**Affiliations:** 1 Research Partner of the Institute of Nanotechnology, Karlsruhe Institute of Technology (KIT), POB 3640, 76021 Karlsruhe, Germany; 2 COSMOS-Software, Johann-Griesbach-Str. 26, 07743 Jena, Germany; 3 Institute of Quantum Optics, Ulm University, Albert-Einstein-Allee 11, 89081 Ulm, Germany; 4 Institute of Nanotechnology, Karlsruhe Institute of Technology (KIT), POB 3640, 76021 Karlsruhe, Germany; 5 Helmholtz Institute Ulm (HIU) for Electrochemical Energy Storage, Helmholtzstrasse 11, 89081 Ulm, Germany

**Keywords:** molecular dynamics MDOC, conformers, RDC, NOE distances, 3J couplings

## Abstract

To study the flexibility of strychnine, we performed molecular dynamics simulations with orientational tensorial constraints (MDOC). Tensorial constraints are derived from nuclear magnetic resonance (NMR) interaction tensors, for instance, from residual dipolar couplings (RDCs). Used as orientational constraints, they rotate the whole molecule and molecular parts with low rotational barriers. Since the NMR parameters are measured at ambient temperatures, orientational constraints generate conformers that populate the whole landscape of Gibbs free energy. In MDOC, structures are populated that are not only controlled by energy but by the entropy term *TΔS* of the Gibbs free energy. In the case of strychnine, it is shown that ring conformers are populated, which has not been discussed in former investigations. These conformer populations are not only in accordance with RDCs but fulfill nuclear Overhauser effect (NOE)-derived distance constraints and ^3^J_HH_ couplings as well.

## 1. Introduction

In the realm of structural biology, and especially in the search for new pharmaceuticals, it is often essential to know the conformational states of molecules in a solution [1]. In the case of small molecules, only one of a multitude of conformers might take part in a reaction—a process that is called conformational selection. The “a priory” prediction of conformer equilibria using computational methods is a formidable task because the Gibbs free energy landscape of the system involves the molecule with its surrounding solution at the temperature of interest [2]. That means we have not only to know the enthalpy Δ*E* of the system but the entropy term *T*Δ*S* as well. This task can only be performed using molecular dynamics (MD), introducing time as the fourth dimension into structure investigations.

Augmenting MD simulations with experimental NMR results was a promising way to gain real insights into the conformational landscape of molecules. The use of residual dipolar couplings (RDCs) proved to be highly attractive because they reflect the time evolution of orientations of molecules and their mobile groups [3,4]. In the papers of De Simone et al. [3,4], a scalar parameter θ derived from RDC was introduced, and this parameter accounts for molecular orientations.

The RDCs are downscaled dipolar interaction tensors of two nuclei, and it turns out that the tensor elements encode molecular orientations: the principal values depend on the orientation of the interaction line of the two nuclei to the magnetic field and the off-diagonal elements on the rotation about this axis. In the recently developed Molecular Dynamics with tensorial Orientational Constraints (MDOC) method [5], all elements of RDC tensors are consequently used as constraints that drive molecular reorientations. One aim of this paper was to show that, for a well-studied system, MDOC simulations can reflect conformations’ equilibria that are present in NMR investigations of solutions. 

**Figure 1:**
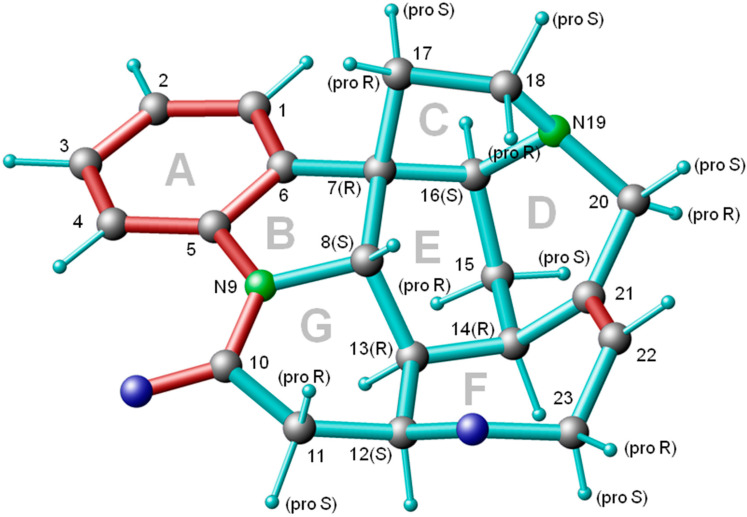
Structure of strychnine [8] with carbon and nitrogen labeling. For CH_2_ groups, the pro-chiral assignment is given. Conjugated π-bonds and double bonds are indicated in red color.

From the early days of the use of RDCs, new techniques in this field have been tested on strychnine [6,7]. These first investigations treated strychnine as a stiff entity because of its bridged ring systems (see Figure 1). Since the structure and chiral configuration were determined by *X*-ray investigations [8], strychnine represents an ideal test case for methods such as MSpin [9] or Pales [10] involving alignment tensors.

The first evidence for the second conformer of strychnine came from precise nuclear Overhauser effect (NOE) measurements of interproton distances of the dissolved strychnine in solution [11]. From inspecting the NOE-measured distance between H11(proR) and H23(proR), Butts et al. [11] concluded that there should exist another low-populated conformer with a different position of the CH_2_ group of the F ring that leads to a shorter average distance compared with the crystal structure [8].

Schmidt et al. [12] gave the first estimate for the population of the second F ring conformer, called the minor conformer. The authors combined Density Functional Theory (DFT) calculations with low-temperature ^1^H-NMR investigations. From the intensity of the second resonance of H22, the authors estimated a population of 5.9% at 298 K from a measurement at 210 K. 

Later, Kolmer et al. [13] performed a complete reinvestigation of the interproton distances and characterized the conformers using RDCs. From inspecting proton distances, the authors arrived at a population of 98% for the major F ring conformer (called **1f1**). Although Butts et al. [14] used the incorrect 1/r^3^ model that can lead to unpredictable errors [15], their result is still close to the findings of 97% population. Additional to the F ring conformers, the flexibility of the C ring was investigated. Using only distances, no second conformer of the C ring could be detected. The same holds for an RDC analysis using the Multi Conformer Single Tensor method (MCST) with more than one conformer.

One major drawback of the methods discussed above was the use of a set of fixed structures representing the conformers. These molecular models are mostly obtained by DFT geometry optimization [12]. One step in the direction of overcoming this limitation was achieved by Tomba et al. [16], who performed a special type of NMR parameter-driven molecular dynamics (MD) simulations. The authors combined meta dynamics with replica exchange MD and applied RDC-derived constraints (θ-method). As a rule, regular, unbiased MD simulations are not able to reach the NMR time scale necessary to describe the conformer equilibria in solution. Though the results of Tomba et al. [16] depended on applied bias and the accuracy of the force field, they clearly indicated the presence of three conformers, A, B, and C, with the latter two occurring in low abundance. Their conformer B (4.9%) corresponds to the buckling of the C ring, and the conformer C (0.2%) to an F ring flip.

Critical energy barriers and limited simulation time can lead in traditional MD simulations to improper conformational distributions [17,18]. However, efficient meta dynamics methods have been developed to surmount these limitations. One of the most efficient methods is to add an external potential consisting of Gaussian functions to provide low-probability transitions [19].

In this paper, we apply a molecular dynamics simulation that is driven by tensorial orientational constraints (MDOC) [20,21]. This method does not depend on the conformer at the start but uses tensorial NMR constraints derived from RDCs to drive molecular reorientations and fragment motions [22,23]. Additional to the RDC tensors, isotropic NMR constraints such as NOE distances and ^3^J-couplings are applied [24] as constraints. MDOC proved its applicability in investigations of the structure and orientation of membrane-bound peptides [25]. In this investigation, all NMR data—RDCs, NOE distances, and ^3^J couplings—are simultaneously used to elucidate the conformer composition of strychnine.

## 2. Methods

### 2.1. MDOC Simulations

A prerequisite of MDOC is a molecular mechanics force field that is flexible enough to calculate the relative energies of most organic molecules and provide structures that compare well to diffraction experiments or more elaborated *ab initio* or DFT calculations. The COSMOS (Computer Simulation of Molecular Structures)-NMR [26,27] force field that was used in this case has one distinct advantage over most other force fields; it uses partial atomic charges from a quantum chemical method [28,29] (Bond Polarization Theory (BPT)) to calculate the electrostatic energy. Since these charges can be recalculated in the course of an MD simulation, all mutual polarizations can be included in the electrostatic energy.

For performing MDOC simulations, a new type of pseudo-forces is introduced into a molecular mechanics force field COSMOS-NMR that causes tumbling and rotations of the entire molecule or its parts. These new orientational pseudo-forces are derived from NMR interaction tensors using all tensor elements as constraints (for details, see Sternberg et al. [5]). The actual interaction tensors calculated at every time step cannot be used as constraints but only their orientational average. In our case, this average is calculated, including an exponential memory function, using a recursion formula (see the SI of the paper by Sternberg et al. [30]). 

In the case of flexible molecules, a multitude of rotamers and transient structures are possible, and these systems cannot be simulated solely with a limited number of RDC tensorial constraints. Because of the ambiguity of the RDC constraints, it is, in most cases, necessary to introduce NOE distances or ^3^J-couplings as additional constraints. This technique was described and was successfully applied to sagittamide A [30] to elucidate the configuration of the molecule and describe the rotamer population in solution (for parameter settings, see Supplementary Material Table S1).

All MDOC simulations are performed using a single molecular model, including all atoms, and their interactions with surrounding molecules are introduced by NMR constraints. Explicit hydrogen atoms have to be implemented, requiring a short MD step of 0.5 fs. Using the COSMOS-Backend for Linux the 160 million steps for the 80 ns simulation required 25 h 49 min on a computing cluster. 

### 2.2. ab Initio Calculations

All calculations were conducted on the level of second-order perturbation theory (MP2) [31] using triple-zeta valence plus polarization (TZVPP) basis sets on all atoms [32,33]. For geometry optimizations, we applied the resolution of the identity approximation of the MP2 method (RI-MP2) [34]. The calculations were carried out using the TURBOMOLE 7.1 software (Karlsruhe, Germany) [35] installed on the bwUniCluster of KIT.

## 3. Results

### 3.1. NMR Data

To keep our simulations as consistent as possible, we used as constraints NOE distances and ^3^J_HH_ couplings data published by Kolmer et al. [13]. Additional to the RDC values (published by Thiele [36]) that were used as orientational constraints, the authors published 33 NOE distances and 13 ^3^J_HH_ couplings. To use these scalar constraints as well in the MDOC simulation, only minor modifications were necessary. Since some of the ^3^J_HH_ couplings were not readily assigned by Kolmer et al. [13], preliminary MDOC simulations with RDC values and NOE distances as constraints were performed, and the ^3^J_HH_ couplings were predicted as mean values over the trajectories. As presented in Table 1, some data revealed characteristic deviations between MDOC simulation and the experiment that could be removed by reassigning (interchanging) the couplings between H18b and H17a/b, as well as the couplings between H12 and H11a/b. In the final MDOC simulation, the 13 ^3^J_HH_ couplings, including the improved assignment, were used as constraints (for the full set of data, see Supplementary Material Tables S2–S4).

Kolmer et al. [13] applied the method of Butts et al. [11] for determining precise NOE distances, but the errors presented for the NOE distances were computed using an extremely low value of 0.003 Å as the variation of the calibration distance. The authors used a distance of 1.760 Å of the geminal protons H15a–H15b as calibration distance; however, this value is 0.02 Å shorter than the calibration standard of 1.780 Å used in many publications (e.g., Kessler & Seip [37]). This difference casts some doubts on the errors presented by Kolmer et al. [13] that are, in some cases, one order of magnitude smaller than the error estimates given by Butts et al. [14] for their method of NOE distance calculations; distances lower than 2.8 Å can be determined with an error 0.05 Å and longer distances up to 4.5 Å with an error of 0.11 Å. These estimates are used throughout this paper despite the problem that for experimental values around 2.8 Å, the error jumps from 0.05 Å to 0.11 Å.

If spin diffusion cannot be experimentally suppressed [38,39,40], simple theoretical models do not describe the NOE intensity well. The calculation conformer fractions from the selected NOE distances may therefore display an additional error. In the case of small molecules, there is a possibility for such situations where the effective distances of a conformer appear smaller than they would be without diffusion, i.e., the constraint MD would overestimate the population of the conformers with smaller distances. 

### 3.2. MDOC Simulated NMR Data

The duration of the final MDOC simulations was 80 nanoseconds at an average temperature of 306.3 K (the details of the setup of the MDOC simulation are presented in Supplementary Materials Table S1). As indicated by the quality parameters *n/χ^2^* > 1 given in Table 2, the NMR data were, on average, correctly reproduced. The *χ* parameters are calculated in the following way:

(1) $$\chi^{2}={\sum_{i}^{n}\left(\frac{q_{i}^{\exp}-q_{i}^{calc}}{e_{i}^{q}}\right)}^{2}$$

In this equation, the *e^q^* denotes the errors of the experimental properties, and a quality *n/χ^2^* larger than 1.0 means that the calculated quantities *q^calc^* are, on average, within the experimental error ranges. In this case, all or at least most summands in eq. 1 are lower than 1.0. As can be seen in Table 2, for the RDC values, we observed two, and for the NOE distances, four outliers. With respect to the ^3^J_HH_ couplings, the simulation gives a perfect result but only if the uncertainty of the Altona equation (prediction error 0.6 Hz) is added to the experimental error (0.65 Hz). If we take the experimental error, only three values would be calculated outside the experimental error range (see Supplementary Materials Table S4).

Let us now have a look at the largest outliers as indicated with a minimum of (*1/χ_i_*)^2^ < 1 (see Table S2 in the Supplementary Material for the full data set): for the H14–C14 RDC, the MDOC simulation provided 30.72 Hz whereas the measurement gave 31.50 Hz. Since the error was estimated to be 0.5 Hz [36] for all RDC values, the calculated value is outside the error range. In the case of the NOE distances, the maximum outlier is the mean distance from H12 to H23a with 2.308 Å. The experimental distance is 2.232 Å, and because of the error of 0.05 Å, the simulated mean value is outside of the error range. The deviations of the other three outliers are smaller or, in one case, negligible.

### 3.3. Conformational Analysis

**Figure 2:**
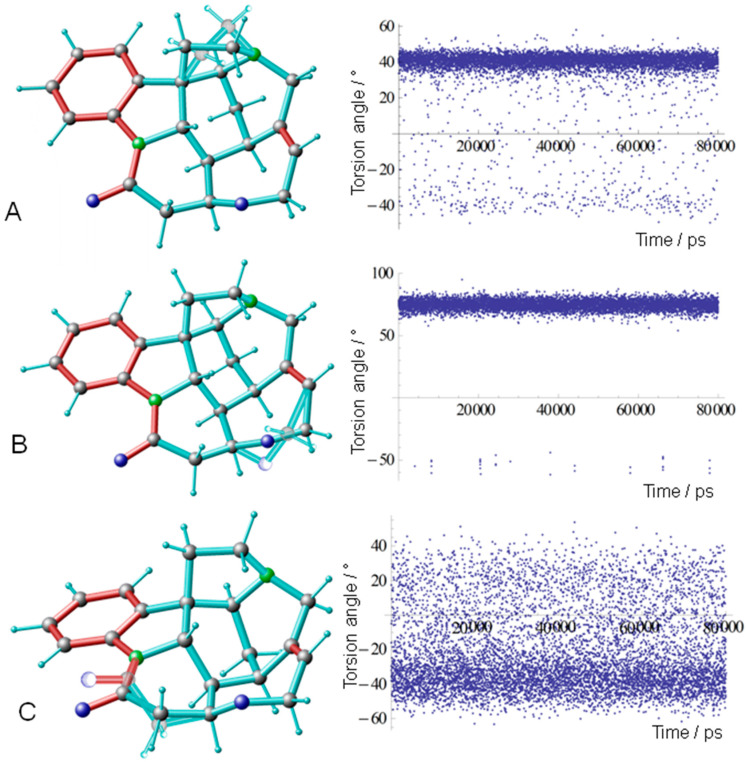
Torsion angle trajectories of the C, F, and G rings. (**A**) Torsion trajectory of the C ring C7-C17-C18-N17 with the major conformation of the C ring **1c1** around 40° and the minor conformation **1c2** around −40°. The minor conformation **1c2** is displayed transparently within the molecular scheme on the left side. (**B**) Torsion trajectory F ring C12-O24-C23-C22 with the major conformation of the ring **1f1** around 75° and the minor conformation **1f2** around −50°. (**C**) Torsion trajectory G ring N9-C10-C11-C12 with the major conformation of the ring **1g1** around −40° and the minor conformation **1g2** in the range between 0 and 50°.

During the MDOC simulation, snapshots of the dihedral angles containing all heavy atoms were saved in all 80 ps producing 39 trajectories containing 8000 dihedrals. Any occurrence of a second ring conformer becomes obvious as a second trace within the trajectory. Figure 2 presents these torsion trajectories of the C ring with dihedral angel{C7-C17-C18-N17}, of the F ring {C12-O24-C23-C22}, and the G ring {N9-C10-C11-C12}. Applying the nomenclature of Kolmer et al. [13], the major ring conformation of the C ring is indicated with **1c1** and the minor conformation with **1c2,** the major F ring conformation with **1f1,** and the minor conformation with **1f2.** Both minor conformers **1c2** and **1f2** show up in the MDOC trajectories as low-populated traces (see Figure 2A,B).

As a novel result, the G ring also displays conformational variability, but this G ring flip angle (Figure 2C) spans a wide range between 0° and 40°. In the major conformation of the G ring **1g1,** the carbonyl group C10 = O10 is, with respect to the view in Figure 4, below the plane of the π system of the benzyl ring, including N9, and flips in the minor conformation **1g2** into a position above this plane.

Besides predicting the likelihood of certain conformers from the MDOC torsion angle trajectories, we can also extract dependent probabilities for the appearance of combinations of torsion angles (Table 3). The largest probability is calculated for the conformer at MDOC, starting with 76.5%, and this is followed by conformers of the G ring with 18.5%, and this conformer is weakly coupled to 1% with the minor conformer **1c2** of the C ring. Disregarding the coupling contribution, the minor C ring conformer has a probability of 3.6%, and finally, taking all contributions together, one obtains 4.7% (also see Table 3).

The occurrence of a minor C ring {−gauche, +gauche, +gauche} conformer was also investigated by Kolmer et al. [13] studying the NOE-determined distance between H18(proR) and H20(proS). The total score function containing all measured NOE distances did not improve by an admixture of the conformer model **1c2.**

**Figure 3:**
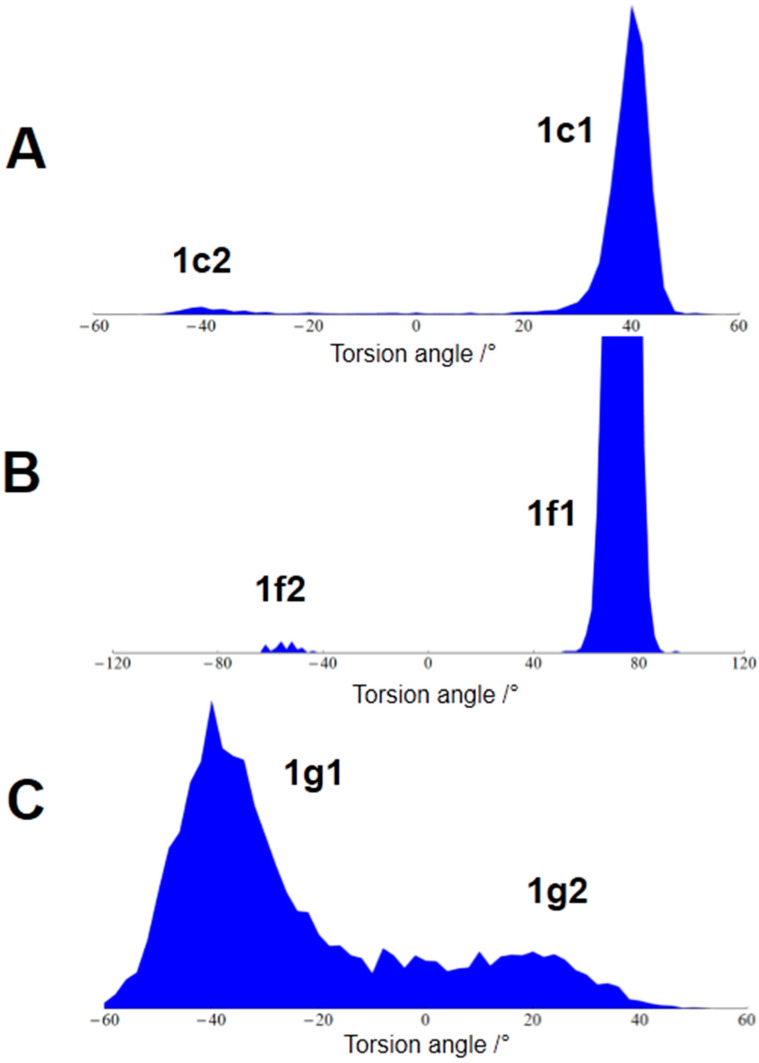
Torsion angle probability distributions for the ring conformations derived from the trajectories in Figure 2. The total probability for the distributions adds up to 100%. (**A**) Torsion angle distribution for the C ring. The probability of minor conformation **1c2** amounts to 4.7%. (**B**) Torsion angle distribution for the F ring (**1f2**: 0.4%). The **1f1** maximum is cut off to show the minor **1f2** contribution. (**C**) Torsion angle distribution for the G ring (**1g2**: 19.6%).

From the investigation of ^1^J_CC_ couplings constants of C ring carbons, Bifulco et al. [41] estimated a very small contribution (0.11%) of the **1c2** structure. In the MD simulations of Tomba et al. [16], conformer B (see Table 4) was observed with a probability of 4.9%. This value corresponds well with the probability of 4.7% observed in our MDOC simulation (see Figure 3). 

Schmidt et al. [12] recorded low-temperature ^1^H-NMR spectra of strychnine (and its protonated form) and investigated the signal of H22 for both conformers. From the intensity of the signals at 210K, the authors extrapolated a contribution of 2.7% at 298K. Kolmer et al. [13] reinvestigated the proton distances supporting the results obtained by Butts et al. [11], and the NOE distance between H11(proR) and H23(proR) (distance from H11b to H23a in [13] was used to investigate the conformers of the F ring. As in the case of the C ring, the conformation of the F ring was estimated by a weighted average of two structure models with **1f1** {+gauche, +gauche, −gauche} and **1f2** {+gauche, −gauche, −gauche} conformation. The total score function revealed a contribution of 98% for the major contribution **1f1** and 2% for the **1f2**. In contrast to these results, the MD investigation of Tomba et al. [16] produced only a small contribution of the **1f2** conformer (called conformer C in [16], see Table 4) of 0.2%. This contribution corresponds better to our MDOC result of 0.4% than the other investigations (sum of the last two rows in Table 3).

Taking these results together, our MDOC simulation confirmed the existence of a second C ring conformer **1c2** and an F ring conformer **1f2** in solution, but the populations compared well only to the MD results of Tomba et al. [16]. As we can see from Figure 2 and Figure 3, the torsion angle of the C ring at ambient temperature spreads out with low probability between the major maximum at about 40° and the minor peak at about −40°. This situation cannot be well described by assigning the NMR data to two fixed structure models. The situation concerning the F ring conformers is different since the MDOC simulation showed a relatively sharp but weak trace at about 50° (see Figure 2 and Figure 3). As can be seen, the focus on one single distance between H11(proR) and H23(proR) leads to an overestimation of the **1f2** conformer population [13].

Geometry optimizations on the MP2 level (TZVPP basis set) are performed for the *X*-ray structure and the **1c2** (C ring flip) and **1f2** (F ring flip) structures. A calculation of the force constants revealed that on our level of approximation, all three structures have, in fact, minimal energy, and the **1c2** structure is about 17 kJ/mol above the *X*-ray structure and the **1f2** structure 9 kJ/mol. 

**Figure 4:**
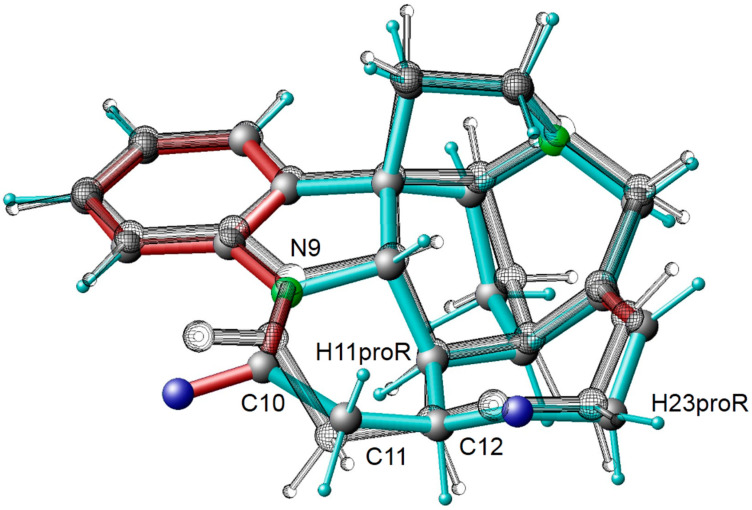
Overlay of two strychnine models with different G ring conformers. In color, the *X*-ray start structure with the torsion angle N9-C10-C11-C12 of −41.7° and in black, the one of the G ring conformers with a torsion angle of 35.2°.

Our MDOC simulations revealed one new feature: the G ring displays conformational flexibility as well (see Figure 3 and Figure 4). In the case of the major G ring conformer (see Figure 4 colored scheme) present in the *X*-ray structure, the N9-C10-C11-C12 torsion angle displays a negative value around −42° (**1g1**). In the situation as displayed in Figure 4, the carbonyl group C10 = O10 is in a position below the plane of the nearby aromatic system. In the course of the MDOC simulation, the N9-C10-C11-C12 torsion angle flips frequently to positive values (see Figure 2 and Figure 3), and a multitude of conformers is generated. In contrast to the C and F ring flips, there is no well-defined maximum between 0° and +40°. We started an *ab initio* geometry optimization with a molecular model selected from the MDOC trajectory with a torsion angle of about 30°, and the procedure ended near the −40° maximum of the major **1g1** conformer (also see Table 4). There is obviously no energy minimum that could lead to a stable **1g2** conformer. All structures between 0° and +40° N9-C10-C11-C12 torsion angle (see Figure 3) are dominated by entropy and are activated by thermal motion. In MD simulations running at ambient temperatures, the entropy term TΔS comes into interplay, and conformers with torsion angles larger than 0° can be observed (see Table 4 conformer B presented by Tomba et al. [16]). 

As Kolmer et al. [13] discussed, the experimental distance of H11(proR) to H23(proR) of 3.596 ± 0.039 Å (see Figure 4) could only be explained by the presence of a second F ring conformer. The H11(proR) to H23(proR) distance of the *X*-ray structure (**1f1**) is 4.2098 Å outside the experimental error margins. The experimental value was therefore regarded as the mean distance of the two conformers, **1f1** and **1f2**. The authors calculated the **1f2** population of 2% from a total score function by adding the distances of the second F ring conformer (**1f2**) to the distance data of a **1f1** molecular model. Since H11proR is connected to the G ring (see Figure 4), the **1f2** population estimate previously performed by Kolmer et al. [13] can only be valid if no second conformer of the G ring is present.

## 4. Conclusions

Many molecules exhibit several shallow energy minima, especially if rotations about single bonds are involved. Additionally, the energy barriers between these rotamers can be low enough to be easily surmounted at ambient temperatures by thermal activations. In these cases, the structures are controlled by entropy. In the case of shallow minima, a multitude of different structures are populated and dominated by a gain in entropy. The occurrence of the G ring flip is not induced by an energy minimum but exclusively by a gain in entropy leading with rising temperature to a more negative Gibbs free enthalpy because of the *−TΔS* term. 

These thermally activated structures lead in regular solution NMR experiments to averaged experimental parameters. Introducing such mean molecular NMR parameters as constraints into molecular dynamics simulations inducts an increased conformational space accounting for the Gibbs free energy rather than energy minima. Tensorial orientational NMR constraints (MDOC) induce realistic molecular motions, and in combination with scalar constraints, one can introduce the influence of molecular surroundings as solvent molecules. In the case of strychnine, it could be demonstrated that the generated conformers are in accordance with 68 NMR constraints. 

The conformers of biomolecules and pharmaceuticals could represent their active states, and it could be of crucial interest to elucidate their structure and reactions. The applicability of this method extends beyond pharmaceutically relevant substances to organic molecules for biomedical or even electrochemical applications. Especially for energy-related materials’ investigations such as organic electrodes or electrolytes, this entropy-driven structure optimization is waiting to be explored.

## Figures and Tables

**Table 1:** MDOC prediction of selected ^3^J_HH_ couplings in comparison to the experiment. The error of the predicted values is estimated to be 1.25 Hz as the sum of the experimental error plus the uncertainty of the Altona equation.

Atom A	Atom B	Predicted ^3^J_HH_ Couplings/Hz	Experimental ^3^J_HH_ Couplings */Hz	Assignment
H18b (pro R)	H17b (pro R)	4.975	10.70	Assigned to H17a (pro S)
H18b (pro R)	H17a (pro S)	12.148	7.20	Assigned to H17b (pro R)
H12	H11 (pro S)	7.902	3.34	Assigned to H11b (pro R)
H12	H11b (pro R)	4.407	8.47	Assigned to H11a (pro S)

* Values obtained from Kolmer et al. [13] (Supplementary Materials).

**Table 2:** Performance of the MDOC simulation of Strychnine.

	RDC	NOE Distance	^3^J_HH_
Number of Constraints n	22	33	13
Number of Outliers	2	4	0
Min (*1/χ_i_*)^2^	0.41	0.43	1.59
Quality (*n/χ^2^*)	2.58	2.61	5.56

**Table 3:** Probabilities for ring conformers of the C, F and G ring and combinations of the conformers. The notation +gauche/−gauche notation is given for the torsion angle combination {C7-C17-C18-N17, C12-O24-C23-C22, N9-C10-C11-C12}.

Probability/%	Notation	Ring Structures	Comment
76.45	{+g, +g, -g}	{1c1, 1f1, 1g1}	Start structure
18.49	{+g, +g, +g}	{1c1, 1f1, 1g2}	G ring flip
3.61	{-g, +g, -g}	{1c2, 1f1, 1g1}	C ring flip
1.06	{-g, +g, +g}	{1c2, 1f1, 1g2}	C and G ring flip
0.36	{+g, -g, -g}	{1c1, 1f2, 1g1}	F ring flip
0.02	{-g, -g, -g}	{1c2, 1f2, 1g1}	C and F ring flip

**Table 4:** Conformers of strychnine collected from the literature.

Model	Torsion Angle C Ring C7-C17-C18-N17	Torsion Angle F Ring C12-O24-C23-C22	Torsion Angle G Ring N9-C10-C11-C12	Conformer
MDOC (76%)	40	75	−40	{+g, +g, −g}
MDOC (18%)	−40	−50	0-50	{−g, −g, +g}
MD conf. A ^1^	31.22	81.71	−42.27	{+g, +g, −g}
MD conf. B ^1^	−24.33	74.62	5.28	{−g, +g, +g}
MD conf. C ^1^	32.17	−69.83	−34.93	{+g, −g, −g}
DFT conf. 1 ^2^	35.93	87.20	−39.16	{+g, +g, −g}
DFT conf. 2 ^2^	33.06	−70.28	−54.59	{+g, −g, −g}
*X*-ray ^3^	38.20	88.20	−41.74	{+g, +g, −g}

^1^ Tomba et al. [16], ^2^ Schmidt et al. [12], ^3^ Glover et al. [8].

## Data Availability

The COSMOS-NMR force field and the routines for MDOC simulations are implemented in the COSMOS-Frontend program (version 6.0) that provides a graphical user interface (GUI) for MS Windows. The computational COSMOS routines for MS Windows and Linux operating systems (COSMOS-Backend) are freely available from the authors (please address requests to the corresponding author, ulrich.sternberg@cosmos-software.de).

## Supplementary Materials

The following supporting information can be downloaded at:https://www.mdpi.com/article/10.3390/molecules27227987/s1, Table S1: Simulation parameters for Molecular Dynamics Simulations with Orientational NMR Constraints; Table S2: Comparison of experimental and MDOC simulated RDC values for Strychnine; Table S3: Comparison of experimental and MDOC simulated NOE distances for Strychnine; Table S4: Comparison of experimental and MDOC simulated 3JHH couplings for Strychnine.
