# Supplementary material for: Structures Controlled by Entropy: The Flexibility of Strychnine as Example
Source: Molecules. 2022 Nov 17;27(22):7987. doi: 10.3390/molecules27227987 (PMC9692940; doi:10.3390/molecules27227987)
Supplement: Supplementary file 1 [file molecules-27-07987-s001.zip › molecules-2003614-supplementary.pdf]

Supplementary Information

Structures Controlled by Entropy: the Flexibility of Strychnine as Example

Ulrich Sternberg and Raiker Witter

Table S1: Simulation parameters for Molecular Dynamics Simulations with Orientational NMR Constraints

General parameters for the MDOC simulations	
Parameter	Value
Target temperature	280 K
MD time step	0.5 fs
BPT atomic charge calculation	0.5 fs
Coupling time η to the heat bath	0.02 ps
Memory decay time τ for the property average	200 ps
Time constant ρ for the exponential rise of pseudo-forces	200 ps
Order parameter of the alignment medium S_{am} (dipolar couplings)	0.01
Total MD duration	80 ns

Width and weight parameters for the pseudo forces in MDOC simulations	
Parameter	Value
Pseudo-force width ΔD for the one bond CH couplings	0.5 Hz
Weight parameter k_D (one bond couplings - in kJ mol^{-1} Hz^{-1})	0.0008
Pseudo-force width ΔR for the NOE distance constraints	0.2 Å
Weight parameter k_R (NOE distances - in kJ mol^{-1} $Å^{-1}$)	3
Pseudo-force width ΔJ for the $^3J_{HH}$ coupling constraints	1.0 Hz
Weight parameter k_J ($^3J_{HH}$ couplings - in kJ mol^{-1} Hz^{-1})	10.0

Table S2: Comparison of experimental and MDOC simulated RDC values for Strychnine

Atom A	Atom B	Experiment[1] RDC / Hz	MDOC Simulation RDC / Hz	Difference Exp.-Sim. Hz
H4	C4	-43,5	-43,183	-0,317
H3	C3	-12,8	-12,645	-0,155
H2	C2	-15,3	-15,177	-0,123
H1	C1	-43,5	-43,16	-0,34
H8	C8	24,6	24,057	0,543[2)]
H16	C16	-14,8	-14,697	-0,103
H18b	C18	-20,6	-20,291	-0,309
H18a	C18	-9,1	-8,779	-0,321
H17a	C17	-25,5	-25,215	-0,285
H17b	C17	13,5	13,333	0,167
H15a	C15	-8,1	-7,713	-0,387
H15b	C15	-2,2	-2,131	-0,069
H14	C14	31,5	30,718	0,782[2)]
H13	C13	12,2	12,068	0,132
H20a	C20	18,9	18,639	0,261
H20b	C20	10,2	10,008	0,192
H22	C22	3	2,964	0,036
H23b	C23	6,3	6,149	0,151
H23a	C23	37,4	36,983	0,417
H12	C12	32,4	32,047	0,353
H11b	C11	33,9	33,683	0,217
H11a	C11	-25,8	-25,748	-0,052

[1] Experiments of Thiele [36] with errors estimated to 0.5 Hz

[2] Outlier

Quality n/χ^2 = 2.58

Minimum $(1/\chi^2)_i$ = 0.41

Table S3: Comparison of experimental and MDOC simulated NOE distances for Strychnine

Atom A	Atom B	Experiment[1] / Å	MDOC Simulation / Å	Error[2] / Å	Difference Exp.-Sim. / Å
H1	H15a	3.734	3.735	0.11	-0.001
H8	H18b	2.147	2.168	0.05	-0.021
H8	H15a	4.22	4.174	0.11	0.046
H8	H13	2.914	2.96	0.11	-0.046
H8	H12	3.48	3.51	0.11	-0.03
H8	H11b	2.536	2.554	0.05	-0.018
H16	H18b	3.78	3.784	0.11	-0.004
H16	H18a	3.611	3.567	0.11	0.044
H16	H15a	2.464	2.472	0.05	-0.008
H16	H15b	2.475	2.458	0.05	0.017
H16	H13	3.872	3.917	0.11	-0.045
H16	H20a	3.32	3.301	0.11	0.019
H16	H20b	3.959	4.001	0.11	-0.042
H18b	H18a	1.691	1.699	0.05	-0.008
H18b	H20b	2.343	2.392	0.05	-0.049
H18a	H20b	2.748	2.734	0.05	0.014
H15a	H14	2.493	2.487	0.05	0.006
H15a	H13	2.188	2.213	0.05	-0.025
H15b	H14	2.423	2.43	0.05	-0.007
H15b	H20a	2.186	2.217	0.05	-0.031
H14	H13	2.251	2.298	0.05	-0.047
H13	H12	2.244	2.301	0.05	-0.057[3)]
H13	H11b	3.788	3.797	0.11	-0.009
H20a	H20b	1.781	1.791	0.05	-0.01
H20b	H22	2.306	2.379	0.05	-0.073[3)]
H20b	H23b	4.774	4.699	0.11	0.075
H22	H23b	2.37	2.424	0.05	-0.054[3)]
H22	H23a	2.858	2.893	0.11	-0.035
H23b	H11b	3.596	3.685	0.11	-0.089
H23a	H12	2.232	2.308	0.05	-0.076[3)]
H23a	H11b	4.254	4.179	0.11	0.075
H12	H11b	2.959	2.942	0.11	0.017
H11b	H11a	1.795	1.788	0.05	0.007

[1] Experiments of Kolmer et al. [13]

[2] Error estimated according the rules as given by Butts et al. [14]

[3] Outlier

Quality n/χ^2 = 2.61

Minimum $(1/\chi^2)_i$ = 0.43

Table S4: Comparison of experimental and MDOC simulated $^{3}J_{HH}$ couplings for Strychnine

Atom A	Atom B	Experiment [1] / Hz	MDOC Simulation / Hz	Error / Hz[2]	Difference Exp.-Sim. / Hz
H8	H13	10.41	10.169	1.25	-0.241
H16	H15b	4.33	3.659	1.25	-0.671
H16	H15a	2.42	2.856	1.25	0.436
H18b	H17b	7.2	6.308	1.25	-0.892
H18b	H17a	10.7	11.122	1.25	0.422
H18a	H17b	3.2	2.451	1.25	-0.749
H18a	H17a	5.5	5.332	1.25	-0.168
H15a	H14	1.96	2.313	1.25	0.353
H15b	H14	4.11	3.86	1.25	-0.25
H14	H13	3.29	2.958	1.25	-0.332
H13	H12	3.3	3.127	1.25	-0.173
H12	H11a	8.47	8.74	1.25	0.27
H12	H11b	3.34	4.332	1.25	0.992

[1] Experiments of Kolmer et al. [13] (Supplementary Information); the assignment of the couplings of H18b(proR) to H17a/b(proR/S) and the assignment of H12 to H11a/b(proS/R) was interchanged.

[2] The error is the sum of the experimental error (0.65 Hz) and the error of the calculation using the Altona equation (0.6 Hz).

Quality n/χ^2 = 5.57

Minimum $(1/\chi^2)_i$ = 1.59
